# Negative pressure ventilation: a special application of expiratory ventilation assistance

**DOI:** 10.1186/s40635-019-0248-z

**Published:** 2019-05-02

**Authors:** Lena Böttinger, José W. A. van der Hoorn

**Affiliations:** Ventinova Medical B.V., Meerenakkerplein 7, 5652 BJ Eindhoven, The Netherlands

To the editor

The recent publication by Berlin et al. evaluated the effect of negative end-expiratory pressure (NEEP) on hemodynamic parameters during ventilation in a porcine model before and after hemorrhage, while NEEP was established using a prototype device with automated expiratory ventilation assistance (EVA) [1]. Compared to conventional volume controlled ventilation with positive end-expiratory pressure (VCV-PEEP) at similar minute ventilation, EVA-NEEP markedly improved hemodynamic parameters in hemorrhagic shock, without the need for massive infusion of fluids. At the same time, the authors noted a lower ventilation efficiency of EVA-NEEP compared to VCV-PEEP, as carbon dioxide (CO_2_) removal was reduced under the conditions chosen, while oxygenation was maintained.

We feel it is worth pointing out that the application of EVA ventilation referred to in this study specifically corresponds to the use of EVA in combination with NEEP, which may be indicated in the special situation of hypovolemia to improve venous return and cardiac output, but does not reflect the main intended use of this technology.

The EVA ventilation was initially developed as an emergency technique [2] and has been elaborated into FCV® as an alternative to conventional positive pressure ventilation. EVA/FCV® make use of the Venturi effect to control both inspiration and expiration. Based on continuously measured intratracheal pressures, EVA/FCV® ventilation provides a constant gas flow into or out of the lungs until the peak inspiratory or end-expiratory pressure aimed for is reached.

Ventilation efficacy of EVA-PEEP was evaluated in healthy pigs by Schmidt et al. [3]. Their study demonstrated that during 6 h of ventilation, FCV-PEEP resulted in improved lung recruitment and arterial oxygenation (+ 10%, *P* = 0.002) as compared to VCV-PEEP at similar peak and PEEP pressures, while using a lower minute volume (− 21%, *P* = 0.04) [3].

Thus, the EVA/FCV® technology allows, but is not restricted to, negative pressure ventilation and can even represent a higher efficient alternative to conventional ventilation when using PEEP. Recently, FCV® has become available for clinical use (ventilator Evone®, Ventinova Medical, Eindhoven, the Netherlands) and has been shown to effectively ventilate patients using PEEP [4]. The manufacturer therefore recommends to apply NEEP ventilation only in situations of hemorrhagic shock.

While discussing the reduced ventilation efficiency of EVA-NEEP compared to VCV-PEEP, Berlin and colleagues suggest that this is in line with earlier investigations. They refer to a study by the workgroup of Enk, evaluating the efficiency of EVA ventilation in a pig model of acute hypoxia [5]. However, in that study, the authors used the manually operated emergency EVA ventilator (Ventrain®) in combination with an uncuffed transtracheal catheter. They showed that EVA ventilation is less effective in an open airway, due to suctioning of false air, but is highly effective in a fully obstructed airway [5].

In the present study, Berlin and colleagues make use of a prototype Tritube®, a cuffed, ultrathin ventilation tube sealing the airway to enhance the efficiency of EVA/FCV ventilation. The decrease in ventilation efficiency described here, as demonstrated by slightly reduced CO_2_ removal, can thus likely be attributed to the intentionally set lower peak pressures and the occurrence of atelectasis associated with NEEP.

## Abbreviations

CO_2_: Carbon dioxide
EVA: Expiratory ventilation assistance
NEEP: Negative end-expiratory pressure
PEEP: Positive end-expiratory pressure
VCV: Volume-controlled ventilation

## References

[1] Berlin DA, Manoach S, Oromendia C, Heerdt PM (2019) Automated expiratory ventilation assistance through a small endotracheal tube can improve venous return and cardiac output. Intensive Care Med Exp. 10.1186/s40635-018-0217-y. PubMed PMID: 30627962; PubMed Central PMCID: PMC6326914. 10.1186/s40635-018-0217-y PMC6326914 30627962

[2] Hamaekers A.E.W., Borg P.A.J., Enk D. (2012). Ventrain: an ejector ventilator for emergency use. British Journal of Anaesthesia.

[3] Schmidt J, Wenzel C, Mahn M, Spassov S, Cristina Schmitz H, Borgmann S, Lin Z, Haberstroh J, Meckel S, Eiden S, Wirth S, Buerkle H, Schumann S (2018). Improved lung recruitment and oxygenation during mandatory ventilation with a new expiratory ventilation assistance device: a controlled interventional trial in healthy pigs. Eur J Anaesthesiol..

[4] Schmidt J, Günther F, Weber J, Wirth S, Brandes I, Barnes T, Zarbock A, Schumann S, Enk D (2019) Flow-controlled ventilation during ear, nose and throat surgery: a prospective observational study. Eur J Anaesthesiol. 10.1097/EJA.0000000000000967 10.1097/EJA.0000000000000967 30730422

[5] Hamaekers AE, van der Beek T, Theunissen M, Enk D (2015). Rescue ventilation through a small-bore transtracheal cannula in severe hypoxic pigs using expiratory ventilation assistance. Anesth Analg..

